# “I have never talked to anyone to free my mind” – challenges surrounding status disclosure to adolescents contribute to their disengagement from HIV care: a qualitative study in western Kenya

**DOI:** 10.1186/s12889-022-13519-9

**Published:** 2022-06-04

**Authors:** Judith J. Toromo, Edith Apondi, Winstone M. Nyandiko, Mark Omollo, Salim Bakari, Josephine Aluoch, Rami Kantor, J. Dennis Fortenberry, Kara Wools-Kaloustian, Batya Elul, Rachel C. Vreeman, Leslie A. Enane

**Affiliations:** 1 The Ryan White Center for Pediatric Infectious Disease and Global Health, Department of Pediatrics, Indiana University School of Medicine, 705 Riley Hospital Drive, Indianapolis, IN 46202 USA; 2 Academic Model Providing Access To Healthcare (AMPATH), Eldoret, Kenya; 3 Moi Teaching and Referral Hospital, Eldoret, Kenya; 4 Department of Child Health and Paediatrics, School of Medicine, Moi University College of Health Sciences, Eldoret, Kenya; 5 Division of Infectious Diseases, Department of Medicine, Brown University Apert Medical School, Providence, RI USA; 6 Section of Adolescent Medicine, Department of Pediatrics, Indiana University School of Medicine, Indianapolis, IN USA; 7 Division of Infectious Diseases, Department of Medicine, Indiana University School of Medicine, Indianapolis, IN USA; 8 Department of Epidemiology, Mailman School of Public Health, Columbia University, New York, NY USA; 9 Department of Health System Design and Global Health, Icahn School of Medicine at Mount Sinai, New York, NY USA; 10 Arnhold Institute for Global Health, New York, NY USA

**Keywords:** Children, Youth, Disclosure, Continuity of patient care, Loss to follow-up

## Abstract

**Introduction:**

Adolescents living with HIV (ALHIV, ages 10–19) experience complex barriers to care engagement. Challenges surrounding HIV status disclosure or non-disclosure to adolescents may contribute to adolescent disengagement from HIV care or non-adherence to ART. We performed a qualitative study to investigate the contribution of disclosure challenges to adolescent disengagement from HIV care.

**Methods:**

This was a qualitative study performed with disengaged ALHIV and their caregivers, and with healthcare workers (HCW) in the Academic Model Providing Access to Healthcare (AMPATH) program in western Kenya. Inclusion criteria for ALHIV were ≥1 visit within the 18 months prior to data collection at one of two clinical sites and nonattendance ≥60 days following their last scheduled appointment. HCW were recruited from 10 clinics. Analysis was conducted by multiple independent coders, and narratives of disclosure and care disengagement were closely interrogated. Overarching themes were elucidated and summarized.

**Results:**

Interviews were conducted with 42 disengaged ALHIV, 32 caregivers, and 28 HCW. ALHIV were average age 17.0 (range 12.9–20.9), and 95% indicated awareness of their HIV diagnosis. Issues surrounding disclosure to ALHIV presented important barriers to HIV care engagement. Themes centered on delays in HIV status disclosure; hesitancy and reluctance among caregivers to disclose; struggles for adolescents to cope with feelings of having been deceived prior to full disclosure; pervasive HIV stigma internalized in school and community settings prior to disclosure; and inadequate and unstructured support after disclosure, including for adolescent mental health burdens and for adolescent-caregiver relationships and communication. Both HCW and caregivers described feeling inadequately prepared to optimally handle disclosure and to manage challenges that may arise after disclosure.

**Conclusions:**

Complex challenges surrounding HIV status disclosure to adolescents contribute to care disengagement. There is need to enhance training and resources for HCW, and to empower caregivers to support children and adolescents before, during, and after HIV status disclosure. This should include counseling caregivers on how to provide children with developmentally-appropriate and accurate information about their health from an early age, and to support adolescent-caregiver communication and relationships. Optimally integrating peer support can further promote ALHIV wellbeing and retention in care.

**Supplementary Information:**

The online version contains supplementary material available at 10.1186/s12889-022-13519-9.

## Introduction

Globally, there are approximately 1.7 million [1.2–2.3 million] adolescents living with HIV (ALHIV, aged 10–19), and the majority (88%) live in sub-Saharan Africa [1]. Control of the HIV epidemic in adolescents depends on the successful implementation of strategies to prevent new infections and to improve virologic outcomes among those who are living with HIV [2, 3]. Adolescents have lower rates of adherence to antiretroviral therapy (ART) and retention in HIV care, as compared to older age groups [2, 4, 5]. Their care is complicated by challenges specific to the adolescent developmental stage. For adolescents with perinatal HIV infection, this includes learning of their HIV status, and coping with a stigmatized and complex medical condition, while striving to achieve developmental tasks of identity formation and peer socialization [6–8].

Disclosure of HIV status to adolescents has been associated with improved ART adherence and retention in HIV care [9–11]. Other benefits include improved psychological outcomes and emotional well-being both for ALHIV and their caregivers [11–13]. Despite evidence of benefits associated with disclosure, the prevalence of timely HIV status disclosure to adolescents is low in sub-Saharan Africa [12–15]. Complex factors surround caregiver decision-making on disclosure to adolescents. These include caregivers’ feelings of guilt for the child’s infection; difficulties initiating disclosure discussions with their children; fears of negative outcomes and perceived inadequate preparation to manage them; perceived HIV stigma; and concerns that the adolescent may be too young, cognitively unable to understand the disclosure, or unable to maintain secrecy regarding their status [11, 12, 16, 17].

The World Health Organization recommends HIV status disclosure to school-age children by age 12, through a gradual disclosure process beginning in childhood [18]. While international disclosure guidelines have been developed, few facilities have established site-specific disclosure procedures [17, 19, 20]. Challenges exist in applying current guidance to HIV status disclosure in the context of a specific adolescent and family [19, 20]. Improving disclosure strategies for ALHIV is key to improving ALHIV outcomes, reducing HIV prevalence, and achieving the UNAIDS agenda to end the HIV/AIDS epidemic by 2030 [13, 21].

Limited data exist regarding the impact of challenges surrounding HIV status disclosure on adolescent disengagement from HIV care. To understand disclosure histories; events and challenges before, during, or after disclosure; and whether events surrounding disclosure contributed to HIV care disengagement, we undertook a qualitative approach. We performed an in-depth qualitative analysis of narratives regarding disclosure and care disengagement among ALHIV who had disengaged from care, their caregivers, and healthcare workers (HCWs) who work with ALHIV, in western Kenya. Further, we elucidated, from this sample, perspectives on improving disclosure processes to support optimal care outcomes among ALHIV.

## Methods

### Setting and study population

This qualitative study was performed in the Academic Model Providing Access to Healthcare (AMPATH) in western Kenya [4]. AMPATH is a partnership between Moi University and Moi Teaching and Referral Hospital (MTRH) in Kenya, and a consortium of North American and European academic institutions led by Indiana University, that has enrolled over 165,000 people in HIV care [22–25]. This study took place at MTRH AMPATH Center, in Eldoret, and Kitale District Hospital, in Kitale, Kenya. MTRH AMPATH Center includes both a large pediatric clinic and the Rafiki Center for Excellence in Adolescent Health. Children with perinatal HIV receive care in the pediatric clinic. After disclosure, adolescents at MTRH are transferred to receive HIV care at the Rafiki Center. The Rafiki Center provides comprehensive adolescent-friendly health services to adolescents with and without HIV infection. Adolescents enrolled in HIV care at Kitale District Hospital have clinic visits scheduled during adolescent specific days.

Kenya’s national HIV treatment guidelines outline developmentally appropriate stages of health education for children with perinatal HIV infection, with a recommendation for full HIV status disclosure to adolescents between ages 9 to 12 [26]. Follow-up is recommended at 1–2 weeks after full disclosure, and with subsequent routine clinic visits. AMPATH’s pediatric disclosure protocol calls for the initiation of disclosure for all children above age 10, and full disclosure by age 14 prior to transition of care to an adolescent-specific or adult clinic. At the study clinics, HIV status disclosure to adolescents is supported by a multidisciplinary care team including youth peer mentors who liaise with adolescents, their families, and clinicians.

### Recruitment and enrolment

Participant eligibility, study enrollment, and other procedures have been previously described [4]. ALHIV were recruited if they were ages 10–19 at their last clinic visit; previously in care at either MTRH AMPATH Centre or Kitale; attended ≥1 visit in the 18 months prior to identification for study eligibility; and had not attended clinic ≥60 days past their last scheduled visit. ALHIV and their primary caregivers were traced by community health volunteers and by the study team [4]. Participants were excluded if they were found to have transferred care without any period of disengagement. Adolescents and caregivers were recruited from May 2019 to March 2020. HCWs were purposefully sampled to include a range of providers (including clinical officers, outreach workers, nurses, and social workers) with experience managing ALHIV at diverse clinical sites providing varying adolescent-friendly services in western Kenya. HCW were recruited from October 2018 to January 2019.

### Ethics

All participants provided informed consent. Adolescents who were younger than age 18 provided assent in addition to their primary caregiver’s consent. Strict procedures were in place to avoid inadvertent HIV status disclosure to participating ALHIV. All adolescents were screened for disclosure using a disclosure screening tool and a separate interview guide, which did not mention the term “HIV,” was used for non-disclosed ALHIV [7]. The study protocol was approved by the Institutional Research and Ethics Committee constituted jointly by Moi University College of Health Sciences and MTRH, and by the Institutional Review Board at Indiana University.

### Semi-structured interviews

The approach to in-depth qualitative interviews in this cohort has been described previously [4]. Semi-structured interview guides were used, which had been developed according to the research questions and an adapted socio-ecological framework incorporating the dynamic trajectories of adolescent development, adolescent HIV management, and the care cascade (Supplementary File 1) [2, 3, 6, 7]. Open-ended questions investigated adolescent-specific barriers and facilitators to retention in HIV care, and narratives of care disengagement. A subset of questions specifically queried experiences with HIV status disclosure, and assessed whether issues surrounding disclosure or non-disclosure influenced HIV care disengagement.

Interviews were conducted by trained research staff with extensive experience working with ALHIV and in qualitative interview methods, and fluency in Swahili and English. Caregivers and adolescents were interviewed separately. De-identified interviews were audio-recorded and transcribed, and all translations were independently verified.

### Analysis

A code book was developed and organized based on the research questions and an adapted socio-ecological framework [7]. Analysis of initial transcripts led to the expansion of the code book, and further refinement through discussion by multiple members of the study team. Several transcripts were then coded by multiple independent coders to establish consensus. All transcripts were then independently coded by multiple team members using Dedoose (version 7.0.23, SocioCultural Research Consultants, Los Angeles). This analysis focused on status disclosure to adolescents with perinatal HIV, who made up the majority of adolescent participants. Systematic reports of codes were generated for interrogation of narratives of disclosure and disengagement from HIV care. Challenges surrounding disclosure were assessed, with comparisons across interviews and across adolescent characteristics. Disclosure was considered delayed according to participant narratives and perspectives.

Detailed findings were recorded in memos and discussed among the study team. Through close reading and re-reading of the data, and comparison of findings and of perspectives (adolescent, caregiver, HCW), overarching themes emerged. These were organized around major themes surrounding disclosure and either adherence challenges or disengagement from HIV care. Major themes and supportive qualitative data were discussed among the study team, and consensus was reached on the final analysis. Findings were summarized, and representative excerpts are presented.

## Results

Interviews were conducted with 42 adolescents who had disengaged from care, 34 of their caregivers, and 28 HCW. ALHIV were 62% female and average age 17.0 (range 12.9–20.9). Thirty-nine ALHIV (93%) indicated awareness of their HIV status on initial screening; an additional adolescent independently reported awareness of their HIV status during the in-depth interview (total 40/42, 95%). Caregivers were primarily female (79%), and included 16 mothers, 2 fathers, 4 aunts, 3 uncles, 3 grandmothers, 3 siblings, and 3 guardians. HCW included 11 clinical officers, 8 outreach workers, 5 nurses, 3 social workers, and one psychologist.

Issues surrounding disclosure contributed important barriers to ART adherence and retention in HIV care. Central themes emerged from triangulation of adolescent, caregiver, and healthcare worker perspectives.

“*There are a number of issues but topping the list is about disclosure.” – HCW*

### Delayed disclosure

Barriers to adherence and retention were described in the context of non-disclosure or delayed status disclosure to adolescents.

*“[Prior to disclosure] I used to leave [ART], maybe they could give me, then I go and throw them away. I never wanted to swallow them totally. I was sad and I just said, I better die because I don’t know what is going on.” – 18 year-old female*

One orphaned, non-disclosed adolescent became disengaged after moving to a new caregiver who had no prior knowledge of the adolescent’s diagnosis, and need for treatment.

*“When her mother passed on, that was the last time that she took the drugs and she was young. She did not know anything. She had lived all those years, until I stayed with her and she was found with [perinatal HIV] at 16 years.” – Caregiver of 18 year-old female*

Adolescents reported difficulties coping after delayed disclosure of their HIV status. Some discussed inquiring about their diagnosis prior to disclosure and not receiving answers. Once they ultimately learned about their status, they struggled to accept the diagnosis, leading them to stop taking ART and disengage from care.

*“Because I disclosed to her when she was 15 years old. She became troublesome, she could disappear from home, refuse to take drugs. Whenever she knew it was her clinic appointment, she would wake up and disappear.” – Caregiver of 19 year-old female*

Some kept their non-adherence or non-attendance to clinic secret from caregivers.

*“[After learning my status] around [age] 14 or 15 there… that's where I really started not taking the Septrin. I stopped and whenever mom would tell me, and because I was coming to the clinic alone, she would tell me, you go to the clinic, I would come to town and go to a movie shop and go back home.” – 20 year-old female*

### Caregiver hesitancy and reluctance to disclose

Most caregivers reported inadequate preparation to handle disclosure to adolescents. They reported not knowing how to begin the conversation, and had worries about how the adolescent would react following disclosure. Some caregivers expressed that further counseling from the clinic was needed for questions that they did not feel prepared to address. For some caregivers, much of their hesitancy appeared to relate to unresolved trauma surrounding perinatal HIV transmission. Some had anticipated their children may “blame” them or that disclosure could otherwise harm their relationship.

*“I used to tell him that he was taking medication because he had a chest problem, pneumonia, his chest was bad. Telling him that he was taking medication because of HIV was very difficult. There was a counselor… he really encouraged us to disclose to our children. I used to try but getting to the point of telling him I’d feel… because it was through me that the child got infected, I used to feel pain.” – Caregiver of 13 year-old male*

One example highlights the significant hesitation among some caregivers to disclose. A caregiver described disengaging their adolescent from care given their observations in their own work in a clinic. They had observed poor adherence and viral resistance among non-disclosed adolescents on ART, and chose to disengage from care until they felt the adolescent would be ready for disclosure and initiation of ART.

*“Some children come here and they ask so many questions… So, in my heart I was just telling God, just help me so that he continues being healthy until that time he will start medication, so that he takes medication knowing, I have started medication and I have started it for what reason. And why am I being given this medication. I was called many times by the doctors and they asked me why I was refusing to have the boy put on medication.”* – *Caregiver of 16 year-old male*

HCW described common situations of trying to persuade caregivers to initiate the disclosure process. Ultimately, delays and lack of preparation sometimes resulted in inadvertent, unsupported disclosure to the adolescent, and poor outcomes when adolescents reacted with refusal to take their ART or to come to clinic.

“*When you look at these adolescents, more so those who have parents, it is not easy to disclose. The parents want to shield these children, they don’t want them to be done for disclosure and if they are not disclosed to, they will not come to the clinic, they will not take the drugs because they don’t know why they are being told to come to the clinic.” – HCW*

### Adolescents coping with feelings of being deceived

Deceptions regarding an adolescent’s diagnosis hindered ALHIV from gaining autonomy, and led to difficult experiences when adolescents learned their status. Adolescents who had been told they were on treatment for an alternative condition found it difficult to cope with feelings of being deceived. Some lost hope, while others blamed their parents for non-disclosure, and refused to continue with care.

*“About 2 to 3 years later he now came back to clinic, and he was really sick at that time… because the caregiver used to tell him the drugs he was taking was for chest pain and headache... when he was told about his status, that is the time he became bitter and actually refused to come to clinic.” – HCW*

### Internalized HIV stigma

When ALHIV learned their status, some struggled with having already internalized HIV stigma in school and community settings. As a result, anticipated stigma and fear of discovery by others hindered their clinic attendance. Information reportedly received in school portrayed HIV as a “killer disease” and made the adolescents lose hope in HIV treatment and care.

*“I went to my mom... and told her, ‘teacher said this and this about this disease’. Then she started to explain to me… because it was my first time knowing it, I couldn’t want to listen to that because it was a shock on me. So, I was kind of resistant, annoyed, not wanting to know more. I was like, ‘if you tell me it is a deadly disease then definitely, I am dead.’ So that was what was in mind.” – 20 year-old female*

### Lack of post-disclosure support and mental health burdens

Some disengaged ALHIV described a lack of support surrounding or after disclosure as a factor in their disengagement from care. Some adolescents learned their status on their own, or had unresolved questions after disclosure.

*“I think it is just those post[-disclosure] torture that are bothering me, I have never talked to anyone to free my mind. Like I normally want someone to explain to me how I was infected.” – 20 year-old female*

Some experienced strained relationships with caregivers or HCWs, particularly when caregivers avoided questions from adolescent related to the disclosure.

*“I started thinking and asking my mother, ‘why you never told me it is like this and that?’ and my mother would become very harsh and I was like, ‘I should not have asked her’.” – 20 year-old male*

Others disengaged after negative experiences at clinic surrounding disclosure.

*“I had to leave [clinic] because that was not a good way to approach someone [the way the doctor disclosed]. I left the clinic. I said to myself that I will no longer go there. When my mother told me to come back, I would refuse, even when my grandmother told me to come, I refused. I became very stubborn, ‘I will not go, I will not go, I will not go’.” – 20 year-old male*

Others disengaged in the context of mental health burdens that arose after disclosure.

*“[Disclosure] disturbed her a lot and she was saying, ‘I will no longer take the drugs anymore, if I am to die, let me die.’ She became sick later on and spent whole months without going to school.” – Caregiver of 15 year-old female*

### Reviewing experiences across adolescence

Examining the influence of adolescent age reinforced that these overarching themes acted across adolescent development and trajectories. As detailed above, disengagement histories during early adolescence included barriers for non-disclosed adolescents who were not aware of their care needs or of the purpose of treatment. At disclosure, some struggled to accept the diagnosis or to cope with other effects of delayed or inadvertent disclosures. Older adolescents reflected on ways in which having experienced these challenges surrounding disclosure, or not having sufficient support to cope after disclosure, contributed to ongoing difficulties and disengagement from HIV care.

### HCW approaches and perceived disclosure support needs

HCW described their approaches to disclosure challenges, and areas where further supports for disclosure could facilitate better outcomes, including HIV care engagement.

Early, planned disclosure to prepare the adolescent for responsibility in care.

HCW emphasized the importance of early engagement of caregivers around disclosure. They perceived that beginning disclosure at an early stage facilitates retention.

*“I think at family level, we need to engage the parents or the caregivers to do disclosure as early as possible. When they do disclosure as early as possible, the child will be growing into adolescence when they know they are taking drugs for a long time and they are taking drugs for this reason.” – HCW*

#### Intensive counseling and follow-up

HCW placed importance on intensive follow-up after disclosure, for both the ALHIV and the caregiver. They noted that close follow-up allows for recognition of challenges before they advance to poor adherence or disengagement from care.

*“I should be keeping close contact of these patients. Like now if this patient comes for disclosure today, I should be reviewing this patient every two weeks, not necessarily coming to clinic physically but to make phone calls so that am in contact with them.” – HCW*

#### Need for HCW training in adolescent care and disclosure

Some HCW described needs for further training and structure in supporting HIV status disclosure to adolescents. They described challenges planning the timing for disclosure, and managing possible post-disclosure challenges.

*“There should be some guidelines on disclosure or, stipulated time on disclosure. I have done disclosure to adolescent but you find that this particular adolescent has been taking his or her drugs very well but immediately you disclose, this particular client changes. ‘Why have I not been told? Why me?’ Like there are some families that the children are HIV positive and others are not. ‘Why me? Why not others?’” – HCW*

#### Peer support integrated within the disclosure process

HCW highlighted the important contributions of trained peer mentors in supporting adolescents. They noted that peer mentors can take on greater roles in disclosure support and follow-up for retention. Engaging caregivers in peer support was also highlighted to help them gain skills to support adolescent HIV care.

“*Those parents who grew up through the support groups are those who handle their children very well. Even their children accepted. Those who disclosed while giving their children information immediately they disclose to them, giving them information and information. You even realize that the children are strong.” – HCW*

#### Needs to address hesitation and prepare caregivers for disclosure and post-disclosure dialogue with adolescents

HCW described needs to better prepare caregivers surrounding disclosure. They noted the importance of caregiver-adolescent communication after disclosure to ensure ongoing care engagement.

*“We have to train the caregivers on issues with disclosure… We have to educate at least the caregivers.” – HCW*

#### Addressing HIV stigma, including in schools

HCW described challenges related to stigmatizing messages in school. They found that outdated, inaccurate, and negative messaging surrounding HIV impaired adolescents’ acceptance of HIV diagnoses and understanding of the importance of HIV care engagement.

*“I think teachers should actually [be reached] on how they deliver information. I still insist, because some children are hurting. They come here and they cry once we do disclosure. They know that the teacher said it kills.” – HCW*

## Discussion

Challenges surrounding HIV status disclosure to adolescents—particularly, delays in disclosure, inaccurate explanations for treatment prior to full disclosure, and unmet needs for greater support and adolescent-caregiver communication after disclosure—can have significant impacts on adolescent wellbeing and continuing engagement in care. Understanding narratives of disclosure and disengagement—including from disengaged ALHIV, their caregivers, and HCW—can importantly inform adolescent HIV care and disclosure strategies. Findings from this study suggest that such strategies should include: enhancing provider training, guidance, and strategies for individualized implementation of disclosure guidelines and evidence-based practices; empowering caregivers to share accurate and developmentally-appropriate health information with adolescents before, during, and after full disclosure; reinforcing processes to ensure timely disclosure to adolescents; and enhancing post-disclosure support, including through HCW practices, peer mentor support, and by addressing adolescent-caregiver communication and relationships.

Challenges encountered in HIV status disclosure to adolescents can include adolescents’ negative emotions following disclosure, coping with HIV stigma, and anxiety about the future, all of which may hinder HIV care engagement [8, 10, 27, 28]. Meanwhile, non-disclosed adolescents may struggle with understanding the need to continue treatment without knowing its purpose or importance [29]. When some adolescents are told that their medication is for a separate condition (such as chest pain or headaches), they may ultimately struggle with acceptance of HIV disclosure and coping with feelings of being “lied to,” particularly as they reconcile their diagnosis with pervasive HIV stigma or inaccurate messages about HIV. Previous studies have similarly shown that delayed or deceptive disclosure often results from caregivers’ lack of preparedness to disclose and finding it easier to disguise the illness; fears of being blamed for the infection; and feelings of guilt and the desire to protect the ALHIV from the diagnosis [11, 13, 30]. Focused efforts on preparing caregivers for disclosure, addressing their concerns, and proactively promoting communication, including truthful, developmentally-appropriate explanations of care between caregivers and their adolescents, are important for eventual full disclosure and successful outcomes.

Counseling for caregivers of children and adolescents with HIV should begin early in childhood, with increasing education as developmentally appropriate [17, 18, 26, 31]. Findings from this study suggest that caregivers should specifically be counseled *not* to give deceptive reasons for medication-taking, but rather that they should be proactively counseled from an early age of the child to give age-appropriate but consistent and accurate messages surrounding ART and clinic attendance. Findings also suggest that stronger support for timely disclosure is needed to prevent poor outcomes in HIV care engagement. Disclosure should be recognized as a process, with formalized support, guidance, and processes for age-appropriate understanding from childhood through adolescence, including for stages before and after full disclosure of HIV infection.

HCW in this study pointed to gaps in the implementation of available disclosure guidelines in the setting, including challenges applying existing guidance to individual adolescents and families, and needs for enhanced training for HCW to handle dynamic aspects of disclosure. This finding resonates with previous literature. Challenges applying WHO or national guidance and needs for tailored resources have been detailed in a systematic review of disclosure models and resources [31]. As similarly described in other studies from low- and middle-income countries, limitations in HCW staffing and turnover of staff result in challenges providing continuing support to adolescents and caregivers following disclosure [30, 32]. WHO recommendations for disclosure to adolescents are relatively non-specific, and challenges exist implementing recommendations across healthcare settings and varied social contexts [18, 28, 31]. As such, further strategies or interventions are needed to support timely disclosure.

Providing disclosure training to HCW on a regular and recurring basis, including training in applying guidelines, and tools supporting developmentally-appropriate information-sharing, may help enhance support to adolescents and families throughout the disclosure process. A multipronged intervention including both HCW training and tools such as for disclosure readiness assessments, and a staged disclosure cartoon book, was well-supported by HCW and caregivers in Namibia [33].

In Ghana, a randomized trial demonstrated that a therapeutic communication and personalized interaction to promote disclosure integrated in HIV care visits resulted in increased disclosure at each study timepoint [34]. In Zimbabwe, a community health worker intervention supporting children with HIV and their caregivers with multiple components included assessments of pediatric disclosure and dedicated support for disclosure and discussions, and resulted in improved virologic suppression [35]. Further insights may be gleaned from the Amagugu intervention, a counselor-led home-based intervention supporting disclosure of maternal HIV status to HIV-uninfected children, given some anticipated common barriers driving caregiver hesitancies to disclose [36, 37]. As highlighted in a recent commentary, adaptations of this promising approach may be needed for disclosure to children or adolescents regarding their own conditions [38]. They emphasize the urgent need for studies of interventions for HIV-affected families [38]. The present study describes challenges of poor parent-adolescents communications following disclosure; interventions to improve family communications may be particularly important for this vulnerable group.

This study highlights roles for integrated peer support for both adolescents and caregivers in the disclosure process [11, 39]. Peer support is crucial for providing adolescent-friendly care, delivering HIV education to newly-disclosed adolescents, reducing impacts of HIV stigma, and facilitating improved care engagement [40–42]. It may be valuable to further leverage peer support groups and peer mentors to facilitate pre- and post-disclosure support to both caregivers and adolescents, and to empower peer mentors to lead post-disclosure support and follow-up. There is increasing evidence that adolescent peer support interventions are feasible, practical, and effective to support ALHIV care in LMIC [16, 43]. Directed training to empower peers in lead roles in disclosure and follow-up support is critical and may be sustainable in the setting.

Limitations include that this study examined histories from disengaged ALHIV and did not include comparisons with ALHIV who were retained in care. HCW were, however, included, who spoke to factors which either facilitate retention or contribute to care disengagement. This analysis focuses on disclosure challenges contributing to care disengagement; other factors influencing disengagement are beyond the scope of this analysis. The validity of findings is supported by the inclusion of contrasting perspectives among ALHIV, caregivers, and HCW across a range of clinic sites. Findings from this study are broadly consistent with research from similar settings [44]. The present study builds on this literature by elucidating ways in which challenges surrounding status disclosure contributed to disengagement from HIV care. Because this is a cohort of disengaged adolescents, and some described challenges were experienced earlier in adolescence, it is possible that this group did not benefit from later improvements in disclosure processes that may have been implemented since. An important strength of this study is the investigation of disclosure processes among disengaged ALHIV and their caregivers; inclusion of these groups allows for exploration of factors contributing to disengagement, which otherwise may be challenging to fully ascertain.

## Conclusions

Complex challenges surrounding HIV status disclosure to adolescents contribute to care disengagement. Underlying issues include delays in disclosure, inadvertent or unsupported disclosure, inadequate or unstructured post-disclosure support, and persistent HIV stigma. These are further complicated by inadequate preparation of caregivers to handle disclosure and its possible challenges, and limitations of formal guidelines or existing HCW training to support disclosure. There is a need to empower caregivers to support children and adolescents before, during, and after HIV status disclosure. This should include counseling caregivers on how to provide children with developmentally-appropriate and accurate information about their health and treatment from an early age, and to support adolescent-caregiver communication and relationships. Integrating dedicated supports for caregivers and adolescents into the disclosure process may enhance positive disclosure outcomes among ALHIV and support retention in care.

## Supplementary Information

**Additional file 1: Supplementary file 1**. Semi-structured interview guide for disclosed adolescents in this study. A semi-structured interview guide was developed according to the research questions and an adapted socio-ecological framework incorporating the dynamic trajectories of adolescent development, adolescent HIV management, and the care cascade. The enclosed version was used with disclosed adolescents in this study. Parallel versions were developed for use with caregivers and healthcare workers, following a similar structure. A dedicated guide for non-disclosed adolescents did not include any mention of HIV or any questions that were not relevant to this group (such as questions regarding disclosure experiences).

## Data Availability

The conditions of informed consent and human subjects research ethics and approval prevent us from sharing the full dataset. All relevant data to this study are included. Dr. Leslie A. Enane may be contacted at lenane@iu.edu for any queries.

## Abbreviations

AMPATH: Academic Model Providing Access to Healthcare
ALHIV: Adolescents Living with HIV
ART: Antiretroviral Therapy
HCW: Healthcare Workers
HIV: Human Immunodeficiency Virus
MTRH: Moi Teaching and Referral Hospital
UNAIDS: United Nations Programme on HIV/AIDS
