# Supplementary material for: “I have never talked to anyone to free my mind” – challenges surrounding status disclosure to adolescents contribute to their disengagement from HIV care: a qualitative study in western Kenya
Source: BMC Public Health. 2022 Jun 4;22:1122. doi: 10.1186/s12889-022-13519-9 (PMC9167528; doi:10.1186/s12889-022-13519-9)
Supplement: Supplementary file 1 — Additional file 1: Supplementary file 1. Semi-structured interview guide for disclosed adolescents in this study. A semi-structured interview guide was developed according to the research questions and an adapted socio-ecological framework incorporating the dynamic trajectories of adolescent development, adolescent HIV management, and the care cascade. The enclosed version was used with disclosed adolescents in this study. Parallel versions were developed for use with caregivers and healthcare workers, following a similar structure. A dedicated guide for non-disclosed adolescents did not include any mention of HIV or any questions that were not relevant to this group (such as questions regarding disclosure experiences). [file 12889_2022_13519_MOESM1_ESM.docx]

**Note to Interviewer:** See the *shaded* text for possible probing questions to use as appropriate.

**Disengaged Adolescent Interview Guide - DISCLOSED**

**Greetings and introductions**

Thank you so much for taking the time to contribute to this research study. My name is [*name*], I am one of the researchers doing the study. The goal of this research is to learn from your experiences accessing HIV care. We are interested in what things make it challenging or make it easier to come to clinic and to continue in care. Long-term HIV treatment is challenging, and some adolescents may miss appointments or stop coming to clinic. We want to know more about how to help adolescents stay in care for life-long treatment. There is no right or wrong answer to these questions; we appreciate your openness to talk about your experience. If any questions are unclear, or make you uncomfortable, please let us know. Thank you so much for your perspective.

***- Start recording -***

1. Has a caregiver or family member been supporting you in your HIV care? *(Who?)*

Has anyone come with you to clinic? *(Who?)*

Has anyone else been available to come with you, if needed?

(If no one else has been able to accompany to clinic:) Can you describe any reasons why no one else is able to accompany you? *(e.g. no one else aware of child’s status, no one else with the means or ability to help, no one else apparently willing to help)*

Did you ever come to care by yourself? *(All of the time? Some of the time?)*

Who primarily has (had) been helping you take your ART medications? *(Who?)*

Did you take ART on your own, without supervision?

1. Can you tell me how long it has been since you last came to clinic for HIV care?
2. Can you tell us about your experience in care?

*How did you come to be in care at the clinic?*

*How long had* you *been coming to clinic?*

*Can you tell us about a time that was very difficult for you in* *care? (How did you cope with that experience? Was there anything that helped you?)*

1. During the time that you were attending clinic, what things made it easier to continue in care (or to stay in care)? Or what things helped you continue in care?

What did you or your caregiver need to do in order for you to continue in care? *(e.g. request leave, save money for transport, explain absences from work or school)*

How did you manage the costs of of continuing in care *(related to transportation or to missed work)*?

1. During the time that you were attending clinic, what things made it more difficult to continue in care?
2. Thinking about yourself and your situation, what things helped you to continue in care?

*(e.g. related to your desire to be healthy, or to learn about your health)*

What things made it more difficult for you to continue in care?

*(e.g. related to the your health, attendance at school, disclosure status, any mental health challenges)*

1. Thinking about your family’s situation, what things helped you to continue in care?

*(e.g. support from caregivers or family members, accompaniment to clinic, education about HIV)*

Were there any challenges experienced in your family that made it difficult to continue in care?

*(e.g. financial challenges, lack of disclosure to other close family, family conflict)*

1. Can you tell us about your experience at the clinic?

Can you tell me about an experience at the clinic that you liked?

Can you tell me about an experience at the clinic that you didn’t like?

Was there anything about the clinic did that made it easier to continue in care? *(e.g. support from clinicians or other staff, support from outreach worker or social worker, participation in a peer group)*

Was there anything about the clinic that made it more difficult to continue in care? (e.g. concerns about clinic staff, quality of care, or concerns about confidentiality?)

How was your relationship with the doctor? Or with other clinic staff?

*Were the staff understanding? Did they make you feel comfortable?*

*Did the clinic staff have enough time to spend with you?*

*Did you have to wait for a long time at the clinic?*

1. (If adolescent has/had been in school:) Regarding your experience in school, was there anything that made it easier for you to continue in HIV care? *(e.g. permission from school, support from matron, maintenance of confidentiality)*

Was there anything about your school experience that made it more difficult to continue in HIV care? *(e.g. difficulty obtaining permission from school, anticipated or enacted stigma from peers or from school staff, lack of confidentiality)*

1. Thinking about the community in which you live, where things helped you continue in care?

*(e.g. support from friends, organizations in the community, declining stigma)*

Were there any challenges in the community that made it difficult to continue in care?

*(e.g. lack of social support, lack of jobs or opportunities, stigma, conflicts)*

1. Can you recall a time (or times) when you missed a clinic appointment? What were the reason(s) at that time? Can you describe what happened?
2. Recently, you haven’t had a clinic visit for some time. We hope to learn more about the challenges for you to come to care, because it may help us understand how to improve care services.

Can you tell us what you understand about why you haven’t been in care recently?

Can you describe any challenges *(e.g. related to your situation / the family / clinic / school / community)* that have made it difficult for you to continue in care?

1. Is there anything that could be done to support you to continue in care?

*(Explore potential support related to stated reasons for disengagement.)*

Is there anything that could be done within the family to help you continue in care?

Is there anything that the clinic could do?

Is there anything that the school could do?

Is there anything that the community could do?

1. Sometimes young people find it difficult to continue in care because of stigma, meaning that they are concerned that they will be judged or discriminated against because of their status.

Do you worry about discrimination based on your status?

Have you experienced judgment or discrimination because of your status? Can you give us an example (*from school / from clinic / from your peers*)?

Can you tell us about a time that you experienced stigma at school? (*Explore their response.*)

Can you tell us about a time that you experienced stigma at the clinic, or because you were coming to clinic? (*Explore their response.*)

1. Some young people have faced very difficult experiences in life. I don’t want to make you uncomfortable, but I would like to understand more about difficult experiences that you may have gone through. Remember that you don’t have to answer any questions that you don’t want to.

Have you had difficult experiences in the past that have caused you stress?

Have you experienced severe illness or death of anyone close to you? Such as a parent, caregiver, sibling or other close relative? What about a friend or schoolmate?

Have you ever been severely ill or hospitalized?

Did you learn about your HIV status in a way that may have been traumatizing? For example, did you discover your status on your own, or from others, before your caregiver or doctor could tell you in a planned way?

Have you experienced any conflicts at home?

Have you ever witnessed or experienced violence, or threats of violence?

Are there other experiences that have been traumatic for you, which have caused you stress?

Can you describe how these experiences in the past may or may not still affect you currently? Have any of these experiences had an affect on your ability to continue in HIV care?

1. Sometimes young people find it difficult to continue in care because they are feeling “stressed.” This stress might relate to feeling isolated, sad, or worried, or from remembering difficult experiences.

Can you tell us about a time that it was difficult to come to clinic because of such stress?

Can you describe some of the reasons that you were feeling stressed?

Was there anything that helped you during that time? *(e.g. support from family / peers / clinic staff?)*

Was there any support that would have helped, but that you didn’t have?

1. Can you tell us about how you learned your status? (*When was this? Was there a reason you were told at that time? What was your reaction? How do you feel about it now?)*

Was there anyone that supported you through that time? Or anything that helped you at that time?

How did learning your status affect your care? Did it make it easier or more difficult to stay in care?

1. Do any of your close family members know about your status? Who knows about your status? Who does not know about your status?

If some of your close family do not know your status, does this affect your ability to continue in care?

*How would disclosing to close family affect your ability to stay in care? To take medicines?*

*What about disclosing to close friends? Or to a romantic partner?*

1. Is there anything else that you wish to share with us about your story in care?

Is there anything else that you can share with us about how adolescents could be supported to continue in care?
